# The association between dairy intake and migraine odds among pediatrics and adolescents: A case-control study

**DOI:** 10.22037/ijcn.v15i4.3062

**Published:** 2022-01-01

**Authors:** Shadi ARIYANFAR, Soodeh RAZEGHI JAHROMI, Nasim REZAEIMANESH, Mansoureh TOGHA, Zeinab GHORBANI, Ebrahim KHADEM, Morvarid NOORMOHAMMADI, Zahra TORKAN

**Affiliations:** 1 Department of Clinical Nutrition and Dietetics, Faculty of Nutrition and Food Technology, National Nutrition and Food Technology Research Institute, Shahid Beheshti University of Medical Sciences, Tehran, Iran; 2 Headache Department, Iranian Centre of Neurological Research, Neuroscience Institute, Tehran University of Medical Sciences, Tehran, Iran; 3 Multiple sclerosis Research Centre, Neuroscience Institute, Tehran University of Medical Sciences, Tehran, Iran; 4 Cardiovascular Diseases Research Centre, Department of Cardiology, Heshmat Hospital, School of Medicine, Gilan University of Medical Sciences, Rasht, Iran; 5 Department of Persian Medicine, Faculty of Persian Medicine, Tehran University of Medical Sciences, Tehran, Iran

**Keywords:** Migraine, Pediatrics, low-fat, Dairy, Odd

## Abstract

**Objective:**

Migraine is recognized as a disease with unknown etiology and various pathophysiologic pathways which are not fully understood. Due to the relation between dairy intake and various chronic conditions in children and also the paucity of data on the probable role of dairy intake on pediatrics’ odds of having migraine, this study was designed.

**Materials & Methods:**

The present study was a population-based case-control design that was accomplished in a tertiary headache clinic.290 child (aged from7 to 14 years old) was included in this study. A definite diagnosis of migraine was performed by a neurologist; concerning the 2018 international classification of headache disorder 3 (ICHD3) criteria. Also, demographic and anthropometric characteristics were obtained. In addition, the usual dietary intake of participants was evaluated using a validated semi-quantitative food frequency questionnaire (FFQ).

**Results:**

Those children in the case group significantly had higher age and BMI means (P.value:0.000). In the second regression model, odds of migraine were 48% (OR: 0.52; 95%CI:0.27-1.00) diminished in the second tertile and 53% (OR:0.47;95%CI:0.24-0.92) in the third tertile of low-fat dairy intake (P-trend:0.03). In the fully adjusted model, the achieved migraine ORs were as followings:0.48 (95% CI:0.240.95) in the second tertile and 0.46 (95% CI:0.21-0.96) in the third tertile (P-trend:0.04), respectively. Children with more high-fat dairy intake also consumed higher amounts of energy, pastries, simple sugar, unhealthy snacks, and hydrogenated oil (P<0.05).

**Conclusion:**

This study results proposed that a greater amount of low-fat dairy intake may attenuate the odds of having migraine attacks in pediatrics and adolescents who might be at risk of headache, which can be attributed to the micronutrient and also to the bioactive content of these dietary components.

## Introduction

Migraine is recognized as the first disabling neurological condition, which is characterized by recurring, pulsating, and unilateral headache attacks Goadsby, Lipton (1). Migraine headaches are identified as a prevalent disorder amongst adults, and it has been established as a common phenomenon in school-aged children. The mean prevalence of migraine in children and adolescents is estimated to be 9.1% globally (2). However, the prevalence rate might be different concerning the diagnostic criteria that are used to detect migraine in the targeted age group. A migraine headache can have a potentially negative effect on the quality of life as an incapacitating disease, in pediatrics and adolescents. Impairment in academic functioning, school attendance, and social interactions are all results of migraine headaches in school-aged children (3, 4).

Migraine pathophysiology as a complex neurogenic disorder is still not fully understood. It has been asserted that genetic factors are involved in migraine pathophysiology (5); however, environmental factors like diet and lifestyle-related factors may have been considered as potential risk factors for headaches in migraineurs (6). Moreover, both vascular and neural mechanisms are hypothesized to be involved in this disorder. Although the available evidence does not confirm the migraine exact mechanism, inflammation and vasodilation have been suggested to be involved in the migraine attack's pathogenesis (7).

 Several dietary trigger factors are hypothesized to play an influential role in migraine pathogenesis in pediatrics and adolescence, through various inflammatory mechanisms (6). Earlier investigations proposed that certain food items like chocolate, citrus fruits and cheese might trigger attacks in those children with migraine. Several studies were conducted in terms of the food intolerance in migraineurs, and out of them avoidance from certain food allergens was the center of the debate. Concerning the probable effect of dietary components in migraine patients, some dairy products including processed cheese, whole milk, and ice cream were categorized as foods to be avoided since they might not be tolerated in those individuals with migraine. Consequently, as an alternative, low-fat milk could be considered a safe choice for migraineurs. On the other hand, particular nutrients found in different foods and beverages might indicate a protective role against headaches in migraineurs including magnesium, calcium, phosphorous, and vitamin D (8).

Dairy products are identified as abundant sources of various micronutrients and other bioactive compounds named calcium, phosphorous, magnesium, vitamin B12, vitamin B2, and vitamin D (9). Therefore, adherence to a diet, which is low in dairy intake, can adversely affect health status, particularly in children and adolescents (10). Several investigations have demonstrated a reverse association between adequate dairy consumption and chronic disease risk (11). Recently published research indicated that dairy intake could decrease migraine development through anti-inflammatory pathways since these products are rich sources of antioxidant vitamins and minerals. (8, 12).

While the recent investigations have shed light on the possible impact of dairy intake on reducing the development of migraine headache in those adult individuals with migraine (6, 8, 12), scarce evidence exists about the possible association between having migraine headaches and dietary dairy intake in school-aged children and also adolescents. Also, due to the effective role of dairy in reducing low-grade inflammation (13) and gut microbiome balance (14) It can be hypothesized that higher consumption of these food items can be beneficial in reducing odds of inflammatory-related conditions such as migraine. Available evidence suggests that dairy product consumption is in association with higher micronutrient intake in children and adolescents and can be indicative of healthier eating habits (15). Regarding the importance of adequate micronutrient intake in the regulation of inflammatory pathways and subsequent influence on migraine pathogenesis, dairy intake might be beneficial in pediatric migraine odds. Therefore, this study concentrated on the association between dairy consumption and the risk of having migraine in pediatrics and adolescents.

## Material & Methods

Study participants

This population-based case-control study's purpose was to investigate the association between dietary dairy consumption and the odds of having migraine in 290 children and adolescent participants (aged from 7 to 14 years old). One hundred school-age children with migraine referred to either the tertiary referral clinic of Sina University hospital or a private headache clinic in Tehran province were enrolled for the case group from June to September 2017. All the migraineurs who were included in this study were examined by the same expert neurologist headache-specialist and acute/prophylactic medications prescription was performed if it was required. A definite diagnosis of migraine was made by a neurologist with respect to the international classification of headache disorder 3 (ICHD3) criteria (16). Also, 190 population-based sex-matched healthy students without migraine headaches were selected as the control group from schools. The schools were located in Tehran and included all school-age groups. As the data collection was a time-consuming procedure the school’s staff randomly asked parents of children to attend a visit at school in scheduled hours to take part in the study. More details regarding our recruitments and study design can be found in our previous research. 

The participants were enrolled in this study concerning the inclusion criteria as follows: the age of between 7-14 years old, did not follow a special diet and had not consumed any dietary supplement. In addition, due to the probable sleep disturbances effects on an individual’s dietary habits and obesity (17), only participants with normal sleep duration (e.g. sleeping for about 8-10 hours per day) were enrolled in this study. The participants’ sleep pattern was determined through asking from their parents. Moreover, to avoid from occurring any minor or major alteration in the participants' dietary pattern, those with any symptoms that were indicators of various chronic diseases like diabetes mellitus, cerebrovascular disorders, gastrointestinal problems or having any sort of allergy or food sensitivity, chronic kidney disease, cardiovascular disorders, psychological or neurological conditions (like other subgroups of headaches, epilepsy or manifestation of seizures, multiple sclerosis, attention deficit hyperactivity disorder (ADHD)) were excluded from this study.

 Demographic and Anthropometric Assessments

After considering the inclusion and exclusion criteria a trained nutritionist conducted a structured interview. Demographic and clinical data including age, gender, and history of previous medical conditions were attained at the study onset throughout a face-to-face interview. Parents were requested to answer the questionnaire on the children's behalf to minimize any probable error during data collecting. The evaluation of economic status was based on the health insurance status of participants since health insurance in Iran is positively linked to income level. Students with health insurance were assigned as economically good and those with no health care coverage were placed in the economically poor category. Information about the anthropometric measurements (weight and height) was documented by the expert nutritionist. Bodyweight assessment was accomplished using a Seca digital scale with an accuracy of 100 gr as the participants were in minimal clothing with removed shoes.

 Height was measured using a measuring tape with a precision of 1 cm, at the time that participants were in a standing position without shoes and as shoulders were aligned normally. Body mass index (BMI) was calculated with the division of weight (kg) by the square of height (m2).  

Dietary Assessments

All participants' usual dietary intakes were evaluated applying a semi-quantitative 168-item food frequency questionnaire (FFQ). The FFQ also had been earlier approved concerning validity and reliability in Iran. The questionnaire was administered by an experienced dietitian throughout a face-to-face interview. It included 168 separate food items with defined and standardized portion sizes (18). The participants were requested to report their annual consumption rate on a daily, weekly, monthly, and yearly basis for each food item. Concerning the children's inability to fully recall each question, we asked their parents to cooperate through the interview to minimize the chance of recall bias. All the questions were asked in person from parents, while children were also present during the interview and none of the participants was required to complete the questionnaire on their own or at home. It took an average of 20 minutes for each participant to answer all the provided questions of the FFQ, depending on how much time they needed to think about each question. Due to the obtained answers, the daily intake of consumed portion sizes was converted into grams/ml using household measures.

The Iranian national food composition table was used for the majority of items. For the remaining unavailable items in the Iranian food composition table, USDA food composition databases were used (19) and Nutritionist version 4 (N4) software that was adapted to Iranian food items was utilized, to calculate individual’s calorie and macronutrients intakes. Total energy intake was calculated by summing up the energy content of all consumed food. Mixed food items such as pizza were analyzed, using the usual restaurants’ recipes. The amount of 1-serving was defined for each dairy product as follows: yogurt (including plain yogurt, full-fat yogurt, creamy yogurt, and fruit yogurt): 8 ounces (240 g), milk (including low-fat milk, whole milk, chocolate milk, fruity flavored milk, and coffee milk): 1 cup (240cc), cheese (all types including creamy cheese, processed cheese, and feta cheese): 1.5 ounces (45g), ice-cream: 5 ounces (150g), kashk (whey product) 1 tablespoon (15g), cream: 1 tablespoon (15g), and doogh 1cup (240cc) (20). Additionally, we categorized total dairy intake into low-fat dairy (including low-fat milk (ml/day), low-fat yogurt (ml/day), feta cheese (g/day), and doogh (ml/day)) and high-fat dairy (including high-fat milk (ml/day), high-fat yogurt (ml/day), creamy yogurt (ml/day), creamy cheese (g/day), and kashk (ml/day).

Protocol approval and patient consent

This study protocol was approved by the Iranian Center of Neurological Research, Neuroscience Institute (research number=97-01-54-38173), and also by the ethics committee of Tehran University of medical science (ethical code number: IR.TUMS.VCR.REC.1397-263). Participants' parents were formally notified about this research purpose in detail and also were requested to sign a written informed constantly at this study onset. 

Statistical analysis 

The sample size of the study was based on our previous experience with this design and no statistical power calculation was conducted before the initiation of the study. Data analysis was performed using SPSS software (version 21 SPSS, Chicago, IL, USA). In addition, the Kolmogorov-Smirnov test was performed to determine the normal distribution of variables, after that independent two-sample t-test or Mann-Whitney U test was applied concerning the normal distribution. Categorical variables were compared by the use of the chi-square test. The main variables were stratified into tertiles cutoff points due to the study population. In addition, models of logistic regression were applied to estimate the association between migraine headache and dairy intake. After that, the odds ratio (OR) with 95% confidence interval (95%CI) was calculated for all tertiles, with the first tertile that was introduced as the reference group. The potential confounders were controlled by binary logistic regression models adjustment. Model 1 regression was adjusted for age and gender, and model 2 was controlled for age, gender, weight and BMI, total energy, respectively, and the adjustments for the last regression model were included age, gender, BMI, and also dietary intakes (per day) included total energy, whole grains, a simple sugar, vegetables, fruits, red meat, poultry, fish, butter and cream, and hydrogenated oil intake, respectively. To test linear trends across categories, P for trends was calculated by computing the median value of each tertile of dairy products, and they were considered as a continuous variable. The significance level was considered as a P value less than 0.05.

## Results

One hundred pediatric and adolescent migraine cases (51 female, and 49 male), and 190 healthy controls (91 female, and 99 male) were enrolled in this case-control study. Also, those children in the case group significantly had higher age and BMI (P.value: 0.000). Further family history of migraine was significant between the two groups of the study (P.value: 0.000) [Table1].

An independent sample t-test was conducted to compare the dietary intake of dairy products among children of case and control groups [Table2]. There was no significant difference observed between the daily intake of various dairy products between the cases and controls. However, according to the table children in the control group tended to have a higher mean intake of low-fat milk, low-fat yogurt, and doogh (219.91, 116.06, 130.61 ml/day respectively), while they were lower among cases (190.00, 98.7, and 118.25 ml/day respectively). To add, the mean intake of high-fat yogurt and kashk was likely to be lower in the control group (37.76 and 1.80 ml/day, respectively) in comparison with the subjects in the case group (40.28 and 2.22 ml/day respectively).

Table 3 indicates the participant’s mean daily intakes of dairy products concerning the tertiles of total dairy consumption. Children in higher tertiles of total dairy intake tended to have a significantly higher consumption of low-fat milk, high-fat milk, low-fat yogurt, high-fat yogurt, feta cheese, and doogh (P. value: 0.000). By low-fat milk, we meant 1.5% fat milk, that contained 1g fat, 5g carbohydrate, and 3.4 g protein in each 100 g of the product and for high-fat milk assessment, we considered 3.5% fat milk that contains 3.3 g fat, 4.8 g carbohydrate, and 3.2 g protein. Low-fat yogurt is also considered as 1.5% fat yogurt, with fat, carbohydrate, and protein content of 1.33 g, 7.11 g, and 4.88 g respectively in each 100 g of the product. Full fat yogurt is considered a 3.5% fat product, consisting of 3.67 g fat, 5 g carbohydrate, and 4 g protein in every 100 g of the product. Each 100 g portion size of feta cheese contained 21 g total fat, 4.1 g carbohydrate, and 14 g protein. And finally, the macronutrient content of each 100 ml of doogh drink is reported as follows: 1 g total fat, 2 g carbohydrate, and 1 g protein (20). 

 Participants' dietary intakes are demonstrated in table 4 according to the tertiles of dairy intake. It was observed that those subjects with higher consumption of total or low-fat dairy products tended to have increased energy, vegetables, fruits, and red meat intake. Those who had more high-fat dairy products intake were also indicated to consume higher amounts of energy, whole grains, cakes and pastries, simple sugar, unhealthy snacks, and hydrogenated oil, during they consumed less refined grains (P.value <0.05). 

The associations between having migraine and total dairy, low-fat dairy, and high-fat dairy daily intakes were estimated by the use of three logistic regression models [Table 5]. Although there was no significant association between total dairy intake or high-fat dairy intake and odds of having migraine, it was established that a higher intake of low-fat dairy was accompanied by a reduction in migraine risk even after controlling for confounding factors and also different foods dietary intakes. The median intakes of low-fat dairy were 156.33, 407.79, and 746.00 ml/day in the first, second, and third tertiles of low-fat dairy, respectively. Furthermore, a significant reduction in odds of migraine was observed in both second and third tertiles of low-fat dairy consumption vs. the first tertile, respectively, after considering confounding variables in the regression models. In the second model, which was adjusted for BMI and total energy intake in addition to age and gender, it was indicated that odds of migraine diminished by 48% (OR: 0.52; 95%CI: 0.27-1.00) in the second tertile, and also by 53% (OR: 0.47; 95%CI: 0.24-0.92) in the third tertile of low-fat dairy intake (P-trend: 0.034). After additional controlling for dietary intakes, those who achieved migraine ORs were as follows: 0.48 (95% CI: 0.24-0.95) in the second tertile and 0.46 (95% CI: 0.21-0.96) in the third tertile in comparison with the first tertile of low-fat dairy intake (P-trend: 0.046).

**Table1:** Baseline characteristics of participants

**Variables**		**Case group** **(n=100)**		**Control group** **(n=190)**		**P.value**	
**Gender [n (%)]**	**Girls** **Boys**	51 (51.0 %) 49 (49.0%)		91 (47.9 %) 99 (52.1%)		0.610	
**Age** ^&^ ** (year)**		11.23 (1.89)		9.91 (1.99)		0.000	
**BMI** ^&^ ** (kg/ m** ^2^ **)**		19.86 (3.77)	18.67 (3.37)		0.000	
**Family history of headache [n (%)]**	**Yes** **No**	81(81%) 19 (19%)	106 (55.8%) 84 (44.2%)		0.000	

^&^ These data are presented as mean (Standard Deviation)

P.value was calculated using Independent t-test or chi-square test

**Table 2:** Comparison of Dietary Dairy Products Intake Between Case and Control Groups

**P value**	**Control** Mean ± Standard Deviation	**Case** Mean ± Standard Deviation	**variable**
0.251	210.34±219.91	210.45± 190.00	Low-fat milk (ml/day)
0.571	136.95±58.28	117.70±49.13	High- fat milk (ml/day)
0.276	126.66 ±116.06	128.89±98.87	Low – fat yogurt (ml/day)
0.847	100.84 ± 37.76	113.51±40.28	High – fat yogurt (ml/day)
0.745	12.51 ± 1.80	6.17 ± 1.37	Creamy yogurt (ml/day)
0.178	20.81±15.23	26.21±19.04	Feta cheese (g/day)
0.676	19.32± 6.16	11.86 ± 5.27	Creamy cheese (g/day)
0.552	180.61±130.61	140.53± 118.25	Dogh (ml/day)
0.312	1.94± 1.29	9.08±2.22	Kashk (ml/day)

All data are presented as Mean (Standard Deviation)

P-value was calculated using Independent sample T-test

*An Asian product which is a mixture of water and yogurt

** An Asian product derived from drained sour yogurt or sour milk concentrates, known as a whey source.

**Table3:** Dairy product intakes of participants according to the tertiles of total dairy intake

**Tertiles of total dairy intake**				
**Variables**	**1** ^st^ ** tertile**	**2** ^nd^ ** tertile**	**3** ^rd^ ** tertile**	**P.value**
**Low-fat milk (ml/day)**	87.40 (87.01)	191.23 (142.50)	348.91 (265.90)	0.000
**High-fat milk (ml/day)**	24.60 (51.67)	44.13 (80.33)	96.32 (198.51)	0.000
**Low-fat yoghurt (ml/day)**	50.16 (59.24)	100.43 (94.35)	179.18 (167.43)	0.000
**High-fat yoghurt (ml/day)**	14.17 (30.94)	32.10 (85.16)	69.35 (153.35)	0.000
**Creamy yoghurt (ml/day)**	1.45 (6.87)	0.62 (2.76)	2.89 (17.04)	0.350
**Feta cheese (g/day)**	11.80 (14.13)	13.25 (13.88)	24.54 (32.90)	0.000
**Creamy cheese (g/day)**	5.67 (10.11)	3.90 (9.53)	7.99 (26.08)	0.340
Doogh *(ml/day)	50.39 (55.67)	105.14 (94.41)	222.73 (238.95)	0.000
Kashk**(ml/day)	1.88 (9.23)	1.11 (1.57)	1.83 (2.42)	0.940

P.value was calculated using general linear model

All data are presented as Mean (Standard Deviation)

*An Asian product which is a mixture of water and yogurt

** An Asian product derived from drained sour yogurt or sour milk concentrates, known as a whey source.

**Table 4:** Dietary intakes of participants according to the tertiles of dairy intake

Tertiles							
	1		2		3		
	Mean	Standard Deviation	Mean	Standard Deviation	Mean	Standard Deviation	
Tertiles of total dairy intake							
Total energy (kcal/d)	1893.84	735.23	2195.86	781.98	2684.00	1078.19	0.000
Refined grains (g/d)	326.78	160.68	295.30	137.26	309.42	134.01	0.507
Whole grains (g/d)	58.45	66.88	54.43	68.90	68.11	69.51	0.296
Cakes and pastries (g/d)	26.92	28.20	27.04	31.13	33.33	36.68	0.142
Simple Sugar (g/d)	51.40	67.20	69.88	96.84	68.12	97.30	0.237
Unhealthy snacks (g/d)	28.23	37.37	38.70	79.77	38.45	50.04	0.269
Vegetable (g/d)	198.53	141.07	278.50	207.55	325.38	236.27	0.000
Fruits(g/d)	394.76	295.13	539.51	357.93	609.47	417.80	0.000
poultry(g/d)	12.02	11.06	13.18	10.07	12.95	11.65	0.602
Fish (g/d)	10.83	14.95	11.84	13.42	10.29	9.94	0.694
Red meat (g/d)	31.96	35.10	34.09	28.64	52.76	58.58	0.000
Processed meat (g/d)	8.06	27.83	6.33	8.69	6.56	16.26	0.628
Butter and cream (g/d)	5.55	11.41	7.85	15.47	5.98	11.29	0.953
hydrogenated oil (g/d)	8.64	13.85	12.77	17.66	10.47	16.07	0.561
Tertiles of high-fat dairy intake							
Total energy (kcal/d)	1912.63	555.19	2278.51	983.45	2589.73	1065.01	0.000
Refined grains (g/d)	343.30	154.03	305.33	141.15	282.41	132.20	0.009
Whole grains (g/d)	47.28	43.84	59.20	69.69	74.67	83.78	0.007
Cakes and pastries (g/d)	16.62	18.00	33.24	33.57	37.55	38.03	0.000
Simple Sugar (g/d)	46.72	71.10	60.27	90.11	82.74	98.91	0.005
Unhealthy snacks (g/d)	25.02	29.17	36.69	49.37	43.83	83.20	0.046
Vegetable (g/d)	239.09	192.47	280.18	204.82	284.02	217.04	0.239
Fruits(g/d)	465.14	335.55	537.77	429.90	542.34	336.06	0.281
poultry(g/d)	11.66	9.07	14.30	12.49	12.18	10.87	0.777
Fish (g/d)	10.50	14.42	11.31	12.15	11.15	12.12	0.823
Red meat (g/d)	41.94	41.17	36.35	36.81	40.61	51.93	0.912
Processed meat (g/d)	3.61	6.25	8.33	18.16	9.02	27.01	0.121
Butter and cream (g/d)	4.98	10.60	6.40	11.96	8.02	15.53	0.118
hydrogenated oil (g/d)	8.41	14.21	10.41	14.03	13.11	19.03	0.048
Tertiles of low-fat dairy intake							
Total energy (kcal/d)	2029.81	893.40	2143.00	700.17	2603.45	1076.62	0.000
Refined grains (g/d)	296.47	152.76	322.53	142.48	312.46	138.11	0.496
Whole grains (g/d)	57.39	64.48	58.02	77.37	65.58	63.09	0.389
Cakes and pastries (g/d)	32.35	32.17	26.98	35.54	27.97	28.77	0.382
Simple Sugar (g/d)	61.89	81.38	58.71	81.25	68.87	101.55	0.550
Unhealthy snacks (g/d)	36.36	80.36	33.46	40.71	35.61	47.10	0.953
Vegetable (g/d)	207.24	165.09	271.71	196.40	324.21	233.44	0.000
Fruits(g/d)	424.65	301.19	508.18	345.75	612.08	431.42	0.000
poultry(g/d)	12.83	12.24	12.12	9.89	13.19	10.57	0.781
Fish (g/d)	10.33	13.15	12.23	14.56	10.42	10.78	0.965
Red meat (g/d)	30.75	29.88	33.72	32.54	54.36	58.83	0.000
Processed meat (g/d)	8.50	27.42	5.83	9.19	6.60	16.41	0.530
Butter and cream (g/d)	6.26	13.39	8.72	16.07	4.44	7.40	0.249
hydrogenated oil (g/d)	10.31	16.01	12.59	17.85	9.03	13.80	0.497

**Table 5:** ORs (95% CI) for suffering from migraine according to tertiles of total dairy, low-fat dairy, and high-fat dairy intake

**Tertiles of total dairy intake**							
**Variables **	**1st tertile **		**2nd tertile **		**3rd tertile **		**P-trend$ **
Total dairy intake ¥ (g/ day)		289.85		530.50		906.79	
No. cases/ controls		38/58		31/66		31/66	
Model 1	1		0.68(0.36-1.27)		0.78(0.42-1.46)		0.517
Model 2	1		0.58(0.31-1.11)		0.53(0.26-1.06)		0.093
**Tertiles of total dairy intake **							
**Variables **	**1st tertile **		**2nd tertile **		**3rd tertile **		**P-trend$ **
Model 3	1		0.62(0.31-1.23)		0.54(0.25-1.18)		0.143
Tertiles of high-fat dairy intake							
Variables	1st tertile		2nd tertile		3rd tertile		P-trend$
High-fat dairy intake ¥ (g/ day)		14.13		57.98		252.63	
No. cases/ controls		34/63		32/65		34/62	
Model 1	1		0.91(0.48-1.69)		1.26(0.67-2.37)		0.350
Model 2	1		0.78(0.41-1.48)		0.93(0.47-1.82)		0.957
Model 3	1		0.77 (0.36-1.54)		0.81(0.37-1.73)		0.755
Tertiles of low-fat dairy intake							
Variables	1st tertile		2nd tertile		3rd tertile		P-trend$
Low-fat dairy intake ¥ (g/ day)		156.33		407.79		746.00	
No. cases/ controls		42/55		28/68		30/67	
Model 1	1		0.53(0.28-1.00)		0.60(0.32-1.11)		0.133
Model 2	1		0.52(0.27-1.00)		0.47(0.24-0.92)		0.034
Model 3	1		0.48(0.24-0.95)		0.46(0.21-0.96)		0.046

¥ These value are presented as median

$ P-trend was calculated using logistic regression model by considering the median of each tertile of total dairy/low-fat dairy/high-fat dairy intake as a continuous variable.

1 regression model adjusted for age and gender

2 regression model adjusted for age, gender, BMI, total energy intake

3 regression model adjusted for age, gender, BMI, total energy, refined grain, whole grain, cakes and pastries, simple sugar, vegetables, fruits, unhealthy snacks, poultry, fish, processed meat, red meat, butter and cream, and hydrogenated oil intake

## Discussion

According to our findings children who were in the control group appeared to have a higher intake of low-fat dairy products including low-fat milk, low-fat yogurt, and doogh, and a lower intake of high-fat yogurt, high-fat milk, and kashk, although it was not significant. The median intakes of low-fat dairy were 156.33, 407.79, and 746.00 ml/day in the first, second, and third tertiles of low-fat dairy, respectively which resulted in a reduction in migraine risk. The study’s results demonstrated that after full adjustments for potential confounders and dietary factors, an increase in the low-fat dairy consumption from 156.33 ml/day to 407.79 ml/day, followed by the highest amount of 746.00 ml/day, led to a 54% reduction in the odds of having migraine among children and adolescents, respectively. It also appears that those with a higher intake of high-fat dairy products are more likely to consume some unhealthy foods including cakes and pastries, simple sugar, unhealthy snacks, and hydrogenated oil. These findings highlighted that adequate consumption of low-fat dairy products along with a healthy diet can attenuate odds of migraine in pediatrics. To the best of our knowledge, no previous study had pointed to the possible effect of dairy consumption in reducing the odds of having migraine in the pediatric age group and this is the first study conducted on the association between dairy intake and odds of migraine in a group of children and adolescents.

Recent literature reported a global declining trend regarding dairy consumption in the children population. This is of great significance since it has been expressed that 30 ml reduction of milk intake by children aged 5-18 years, followed by 126 ml increase in sweetened beverage consumption These findings suggest the displacement of dairy products by hypercaloric sweetened beverages among pediatrics. This can result in the manifestation of various chronic conditions such as cardiovascular disorders that have a shared pathology with migraine (21). Also, available evidence declared that Preschool children are recommended to consume 2 servings of dairy products daily (22) and this amount increases to 3 servings a day among adolescents. In this regard, a study by Shokrvash et al showed that the average dairy product consumption in Iranian adolescents is below the recommendation (3serving per day), followed by negative health-related outcomes (23).

This study's findings are at least partially in agreement with the result of a study accomplished by Mirzababaei et al. (8). In this first cross-sectional study, 266 women affected by migraine headaches were investigated. These findings indicated that a 30% fall in the number of severe migraine attacks was achieved in those with higher adherence to the Dietary Approach to Stop Hypertension (DASH) diet (rich in low-fat dietary dairy), along with a significant reduction in the duration of the headache in the same group. Also, severe headaches prevalence (VAS: 8-10) was 46% less in those subjects with the highest adherence to the dash diet in comparison to those with the lowest adherence (OR: 0/54). Moderate headaches (VAS 4-7) also had a 36% reduction in terms of frequency (OR: 0/64). Participants in the highest quartile of dash diet reported higher low-fat dairy products intake (270/47) (8). Also, our results are in accordance with the results of the women's health study, conducted on a sample of 39.876 women.

 Total dairy products intake was all evaluated in the targeted population including cheese, cream cheese, yogurt, skim or low-fat milk, ice cream, frozen yogurt, and cream. Those individuals with migraine reported lower low-fat milk intake, in comparison with non-migraineurs (4/8 ± 0/3 Vs.5/3 ± 0/2 cups/day), however, it was not statistically significant. On the contrary, the diet quality, defined as a diet in line with DGA (Dietary Guidelines for Americans) recommendations to meet all food groups and nutrients with maintaining a balanced energy intake, was higher in non-migraineurs in comparison with migraineurs (52/5 ± 0/9 vs 45/9 ± 1/0; P < 0/0001) According to the study’s report individuals with higher diet quality had consumed higher amounts of total fruits, dark green and orange vegetables, low-fat dairy, legumes and fewer amounts of sodium (24). Additionally, findings of a recent study by Mansouri et al, were in line with our results as it revealed that dairy consumption among university students reduced the odds of primary headaches (25).

The trigeminovascular system activation with respect to the inflammatory agents' incremental level may probably contribute to neurogenic inflammation, which may occur after the release of the vasoactive neuropeptide. Releasing the calcitonin gene-related peptide (CGRP) and P-substance, which are known as pain mediators, might be induced via the ganglia trigeminal vascular nociceptors activating (26). Trigeminal nerves, cerebral cortex, and brain stem could be influenced by different dietary components (6). Dairy has indicated a promising effect in inflammation and oxidation-reduction, 

which is mainly attributed to the existing micronutrient and bioactive components (27). They are considered as numerous micronutrient-rich sources including calcium, phosphorous, magnesium, zinc, niacin, riboflavin, vitamin B-6, vitamin B-12, coenzyme Q10 (28). A diet rich in calcium curbs the inflammatory cytokines, and also contributes to the anti-inflammatory cytokines promotion (32). Findings of a study accomplished by Mirzababaei et al inferred that dairy products consumption may contribute to inflammation through anti-inflammatory pathways as these products are rich sources of calcium (8). This micronutrient imposes anti-inflammatory and anti-oxidative effects in conjunction with 25-dihydroxycholecalciferol that was recognized as an ant-inflammatory factor (29) In addition, the calcium content of dairy plays a restrictive role against inflammatory stress throughout suppression of angiotensin-converting enzyme and α1-25 dihydroxycholecalciferol (29).

The results of one previous study showed that dairy consumption can induce an anti-inflammatory effect through inhibition of nuclear factor κB (30). Some studies claimed that hyperactivity of nuclear factor-κB can promote hypothalamic inflammation which is strongly related to migraine (31). In our study higher amounts of dairy products was associated with a lower risk of having migraine. 

As it was mentioned before, dairy products are also recognized as a source of magnesium. Suppressing the inflammatory cytokines release, and inhibiting platelet aggregation along with vasodilatory effects are amongst those proposed protective roles of magnesium in migraine pathogenesis. Magnesium decreased level may also provoke the release of P-substance. An elevated level of this nociceptive element resulted in a significant increase in the level of the inflammatory cytokine, like tumor necrosis factor - α (TNF-α) and interleukin – 6 (IL-6). 

Magnesium also could alter the CGRP level in circulation. CGRP release Suppression is likely to be effective in the prevention of the attack in migraineurs since elevated levels of CGRP are observed in the jugular vein during a migraine attack. Increased levels of CGRP can induce pain by stimulation of trigeminal neurons and this can be followed by the production of inflammatory cytokines (32-33).

Dairy in particular milk is also identified as an abundant source of B complex vitamins including Riboflavin (B2) and vitamin B12.(34). Migraine pathogenesis has also been associated with an alteration in mitochondrial energy metabolism. Riboflavin has indicated promising effects in the mitochondrial energy metabolism level along with improvements in the complex 1 and 2 activities (35).

More importantly, the interrelationship between gut and CNS, known as gut-brain-axis, might vastly be influenced by the composition of gut microbiota through various mechanisms. Short-chain fatty acids (SCFAs) which are produced by distal colon bacteria can promote gut immunity and minimize inflammations by maintaining gut permeability. The levels of these metabolites can be influenced by dietary items such as probiotics (36). 

It is also noteworthy to state that the Lactobacillus content of fermented dairy products, which is the most frequent probiotic found in yogurt, is capable of promoting gut immunity as they impose inhibitory properties against the inflammation throughout imposing a modulatory role in the level of the pro-inflammatory cytokine-like TNF-α, IL-6, interleukin - 1β (IL-1β). These cytokines are held responsible for the manifestation of migraine pain as they have witnessed to increase during migraine attacks (37). Zemel et al. explained that a significant reduction in inflammatory cytokines (namely: TNF-α, IL-6) was achieved after dairy-based smoothies consumption (38).  

It has been alleged that whole-fat dairy is abundant in saturated fatty acids including butyric, caprylic, and capric, contributing to 95% of the total whole-fat dairy lipid proportion. Medium and longer chain fatty acids are also found in whole–fat milk (lauric, myristic, palmitic) (39). Recent studies indicate that pro-inflammatory cytokines (TNF-α, IL-1, IL-6) productions are remarkably influenced by the high-fat diet that is mainly rich in saturated fat. (40). Ferrara et al. observed in an investigation that adherence to a low-fat diet with a decreased amount of SFAs (12%), led to a noticeable improvement in the migraine headaches status in adults (41) which is partially in agreement with this study findings. Also, we found that those with higher high-fat dairy products intake are more likely to consume some unhealthy foods. This is associated with a higher probability of weight gain in children with ages ranging from 5 to 7 years old (42). With respect to Oakley et al, a relation between migraine and BMI amongst children has been found. This is mainly due to the incremented levels of pro-inflammatory agents (Neuropeptide Y (NPY), CGRP) that are associated with abdominal obesity and blamed to be in association with the pain manifestation in those individuals with migraine (43).

Consequently, these findings along with earlier provided evidence, propose a protective role for low-fat dairy intake towards decreasing the risk of having migraine in pediatrics and adolescents. This study has owned some strengths points along with limitations. Accordingly, the precise estimation of dairy intake in participants using FFQ cannot be fully achieved and as an indispensable part, recall bias is inevitable in a case-control design that occurs systemically. However, several studies have indicated that FFQ is an efficient approach to dietary management in children (44). To the best of our knowledge, in terms of strong points, this study was the first study that has the purpose of investigating dairy intake of pediatric and adolescents with migraine, compared to healthy sex-matched controls. More importantly, migraine can affect various aspects of life in both children and the family. Therefore, investigating underlying nutritional and lifestyle trigger factors, which are mainly modifiable, has been valued. In addition, all the patients with respect to ICHD III criteria were diagnosed by our expert neurologist-headache specialist. Based on this study’s findings a balanced diet with an adequate amount of low-fat dairy products (2-3 servings per day) is recommended for children and adolescents. Our results may provide a rationale for further clinical trials and longitudinal studies on the impact of various types of dairy products on other aspects of migraine headache.

In Conclusion, current study findings proposed that greater low-fat dairy intake in pediatrics and adolescents may attenuate the odds of having a migraine headache. Although more clinical trials are needed to identify the protective role of dairy against migraine, it seems that recommendations for dairy intake might ameliorate the progression of the disease. The observed inverse association between migraine risk and low-fat dairy could highlight the protective role of dairy products against inflammatory pathways that might be mainly attributed to these dietary components, micronutrient, and bioactive content. However, more investigations are needed to strongly declare this recommendation.

## Authors’ contributions

 This study was funded by Iranian Centre of Neurological Research, Neuroscience Institute, Tehran University of Medical Sciences, Tehran, Iran (Grant Number: 97-01-54-38173).

M.T., S.R., Z. G., Sh. A. participated in the design of the study. M.T., S.R., Sh. A., M.N., Z.T. participated in data collection. Z. Gh., N.R. and Sh. A. performed data analysis. Z. Gh., N.R. and Sh. A. contributed in data analysis. M.T., S.R., Z. Gh., Sh. A. and N.R. participated in drafting the article .M.T., S.R., Z. Gh., Sh. A., N.R., M.N., Z.T. and E.Kh. read, edited and approved the final draft.

## Conflict of Interest

The authors declare no potential conflicts of interest with respect to the research, authorship, and/or publication of this article.
